# Maximization of information transmission influences selection of native phosphorelay architectures

**DOI:** 10.7717/peerj.11558

**Published:** 2021-06-10

**Authors:** Rui Alves, Baldiri Salvadó, Ron Milo, Ester Vilaprinyo, Albert Sorribas

**Affiliations:** 1 Ciències Mèdiques Bàsiques, Universitat de Lleida, Lleida, Spain; 2 Plant and Environmental Science, Weizmann Institute of Science, Rehovot, Israel

**Keywords:** Biological design principles, Biological information transmission, Selection, Bacterial signal transduction, Mathematical modelling

## Abstract

Phosphorelays are signal transduction circuits that sense environmental changes and adjust cellular metabolism. Five different circuit architectures account for 99% of all phosphorelay operons annotated in over 9,000 fully sequenced genomes. Here we asked what biological design principles, if any, could explain selection among those architectures in nature. We began by studying kinetically well characterized phosphorelays (Spo0 of *Bacillus subtilis* and Sln1 of *Saccharomyces cerevisiae*). We find that natural circuit architecture maximizes information transmission in both cases. We use mathematical models to compare information transmission among the architectures for a realistic range of concentration and parameter values. Mapping experimentally determined phosphorelay protein concentrations onto that range reveals that the native architecture maximizes information transmission in sixteen out of seventeen analyzed phosphorelays. These results suggest that maximization of information transmission is important in the selection of native phosphorelay architectures, parameter values and protein concentrations.

## Introduction

Organisms and cells use signal transduction circuits to detect environmental changes and make decisions on how to adjust their internal milieu to better survive those changes. Those circuits modulate the cellular response and its metabolic adjustments.

**Figure 1:**
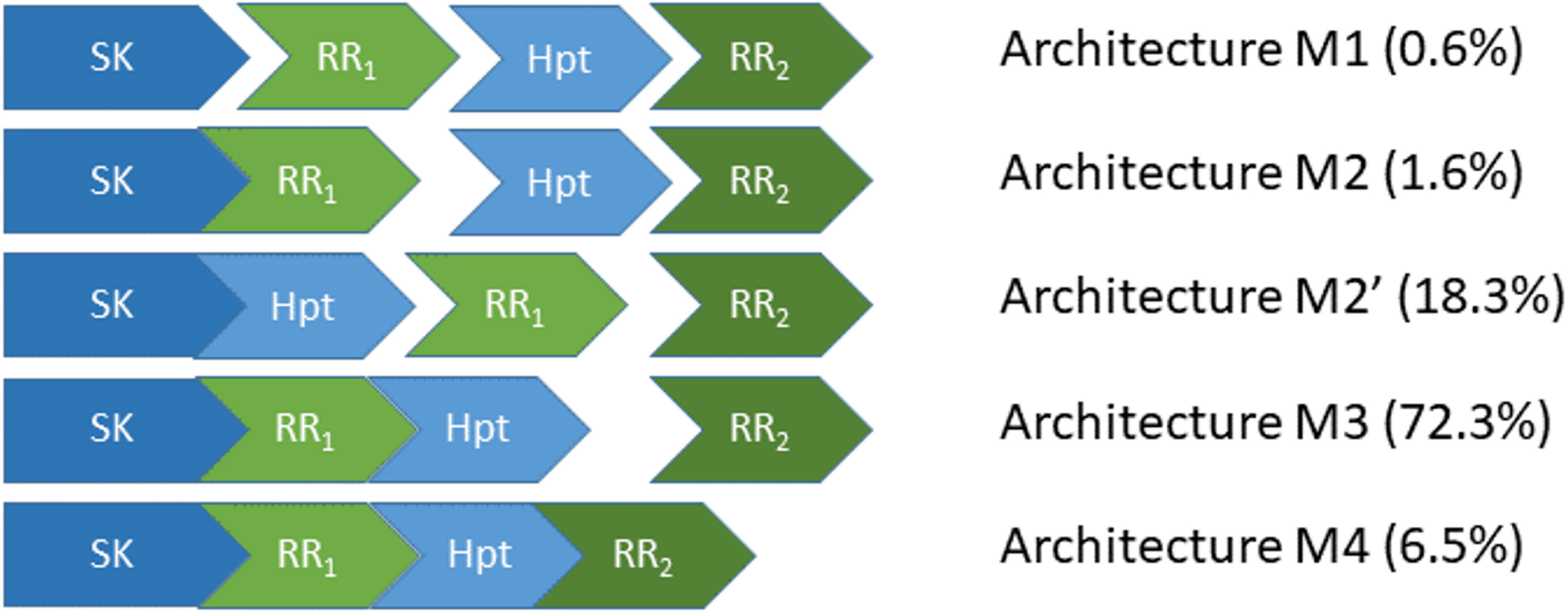
The five most abundant phosphorelay circuit architectures, as inferred from operon structure, account for over 99% of all detected phosphorelays. Architecture M1 is for a circuit where the four phosphorylatable domains exist in independent proteins. Architecture M2 is for a circuit with a hybrid Sensor Kinase (SK), which contains the SK and the first Response Regulator (RR_1_) domain in the same protein, while the remaining phosphorylatable domains exist in independent proteins. Architecture M2′ is for a circuit where the SK and the Hpt domains are in the same protein, while both RR domains exist in independent proteins. Architecture M3 is for a circuit where the SK, RR_1_ and the Hpt domains are in the same protein, while the final RR, RR_2_, is in an independent protein. Architecture M4 is for a circuit where all phosphorylatable domains exist in the same protein. A total of 5219 PR operons were surveyed, out of which 5,182 fall in one of the five architectures shown here.

Phosphorelays (PR) are a large and important class of signal transduction circuits in microorganisms and some plants (Ulrich & Zhulin, 2010; Porter, Wadhams & Armitage, 2011; Kobir et al., 2011; Salvado et al., 2015). A self-phosphorylating Sensor Kinase (SK) modulates its own phosphorylation state in response to the environmental signal. The phosphorylated SK transfers its phosphate to an aspartate residue in a phosphotransfer intermediate protein (RR1). This response regulator transfers its phosphate to a Histidine phosphotransfer (Hpt) protein domain, which then transfers the phosphate to a final response regulator (RR2) (Fig. 1). The phosphorylation state of the circuit modulates cellular activity and adaptation. PR are important for making life or death decisions about sporulation (Burbulys, Trach & Hoch, 1991), in adapting to various stressors (Bekker, Teixeira de Mattos & Hellingwerf, 2006), such as changing levels of oxygen (Georgellis, Kwon & Lin, 2001), or in developmental decisions made by many plants (Kieber & Schaller, 2018).

The four-step nature of the PR phosphotransfer cascade enables the creation of signal-response curves that are steeper than those of two component systems (TCS) (Feliu, Knudsen & Wiuf, 2012; Feliu et al., 2012; Knudsen, Feliu & Wiuf, 2012; Kothamachu et al., 2013; Jovanovic et al., 2015), a simpler signal transduction alternative to PR with only two steps (Alves & Savageau, 2003; Igoshin, Alves & Savageau, 2008; Salvado et al., 2012; Sáez, Wiuf & Feliu, 2017). Steeper response curves are required for cells to mount ultrasensitive, all-or-non responses, such as sporulation in *Bacillus subtilis* (Narula et al., 2012; Narula et al., 2015). Another difference between TCS and PR is that only PR enable reversible phosphotransfer between phosphorylated domains of the signal transduction cascade (Janiak-Spens, Cook & West, 2005; Tindall et al., 2010). These differences might explain the selection of PR as an alternative to TCS signaling.

Still, PR circuits can assemble the four-step cascade using alternative protein domain assembly architectures (Kim & Cho, 2006; Feliu, Knudsen & Wiuf, 2012; Knudsen, Feliu & Wiuf, 2012; Kothamachu et al., 2013; Jovanovic et al., 2015; Salvado et al., 2015) (Fig. 1). On one extreme of the circuit architecture (M4, Fig. 1), all phosphotransfer steps happen in the same protein. In the other extreme (M1 or M2′, Fig. 1), all phosphotransfer steps occur between different proteins. In between, there are other architectures where some phosphotransfer steps happen in the same protein, while others occur between different proteins (M2 and M3, Fig. 1). This raises the question of whether alternative PR architectures may have irreducible physiological differences for the behavior of the circuit or if those differences may be quenched easily by evolving appropriate parameter values (Forst & Roberts, 1994; Samadani, Mettetal & Van Oudenaarden, 2006; Igoshin, Alves & Savageau, 2008; Hazelbauer & Lai, 2010). In this context, can we identify general physiological requirements that dominate the selection of PR circuit architectures and/or parameters?

To understand the physiological differences between the five PR architectures we consider a set of physiological variables (Table 1) that are important for the function of PR and focus on two PR that are kinetically well characterized: Spo0 of *Bacillus subtilis* and Sln1-Ypd1-Ssk1-Skn7 of *Saccharomyces cerevisiae*. We create mathematical models for each system, and identify which architecture performs best with respect to each physiological variable. Of these variables, we found that only information transmission through the circuit was optimized in the natural architecture with respect to all alternative architectures. To investigate how general this result is, we scanned parameter values and protein abundances and identified the regions in parameter space for which information transmission through the circuit is maximized by each architecture. Then, we analyzed seventeen phosphorelays, for which experimental determinations of protein abundance are available but a full set of kinetic parameters is not. The architectures of 16 of these are also consistent with maximization of information transmission.

**Table 1:** Physiological variables used as proxy for performance in signal transduction circuit.

Variables	Circuit performance improves with^a^	Experimental support
Signal amplification	Higher amplification	(Alves & Savageau, 2003; Kærn et al., 2005; Igoshin, Alves & Savageau, 2008; Kuchina et al., 2011; Salvado et al., 2012; Espinar et al., 2013; Berens et al., 2017)
Noise attenuation^b^	Attenuated noise	(Kærn et al., 2005; Kaufmann & Van Oudenaarden, 2007; Raj & Van Oudenaarden, 2008; Tiwari & Igoshin, 2012)
Information transmission^b^	Higher transmission	(Kærn et al., 2005; Kaufmann & Van Oudenaarden, 2007; Raj & Van Oudenaarden, 2008; Tiwari & Igoshin, 2012)
Robustness to changes in parameter values	High robustness (low sensitivity)	(Alves & Savageau, 2003; Igoshin, Alves & Savageau, 2008; Cağatay et al., 2009; Salvado et al., 2012; Berens et al., 2017)
Speed of response to changes	Rate of adaptation	(Alves & Savageau, 2003; Igoshin, Alves & Savageau, 2008; Kuchina et al., 2011; Cheong et al., 2011; Salvado et al., 2012; Narula et al., 2015; Shimell et al., 2018)
Metabolic cost of circuit	Low cost	(Noor et al., 2016; Pereira et al., 2018)

**Notes.**

a As a general trend.

b Relevant in the stochastic domains of dynamic behavior.

Together, the results reported here have consequences for understanding the evolution of PR signal transduction circuits and for the development of synthetic biology PR circuit applications.

## Materials and Methods

### Mathematical modelling

#### Mechanistic models

PR shown in Fig. 1 represent more than 99% of all PR circuits present in the fully sequenced genomes of more than 9,000 organisms. This information was obtained from Salvado et al. (2015).

We used a mass action description of reactions to create a mechanistically more detailed model for each architecture (Alves et al., 2008). These models are given in Appendix S1. They were parameterized with experimentally determined parameter values and protein concentrations compiled from the primary literature. We used these models to create an atlas of performance with information transmission through the PR as a function of protein amount, parameter values, and circuit architecture. The Mathematica notebooks implementing the models and analysis are provided in Supplemental File S5.

#### Estimating protein concentrations

Proteins occupy approximately 20% of cell volume across the tree of life (Dill, Ghosh & Schmit, 2011; Milo & Phillips, 2015). Taking into account average protein sizes, average protein masses and factoring in cell volumes one can estimate that total protein concentration in cells is of the order of ∼1 µM (ranging between 0.4−1.4) (Uri & Milo, 2010), or 6. 23 × 10^6^ proteins per µm^3^. We then use the average cell volume for the different cell types to estimate the total amount of proteins per cell.

In order to estimate biologically relevant protein amounts for each circuit topology we used whole-proteome relative abundance determinations (Table S1) reported by PaxDB (Wang et al., 2015). The database information is given in parts per million. By multiplying these number by total number of cell proteins, we calculate how many proteins exist for each experimental PR system, within the experimental error.

With this information we can further estimate typical orders of magnitude for the ratios between protein abundances within a PR circuit. In general, we find that protein abundances and ratios between protein abundances have a limited range in PR (Table S1).

#### Parameter values for the mass action models

To find experimentally determined parameter values for the individual reactions of the PR, we searched Medline and the Biomodels database (Glont et al., 2018). We searched for previous mathematical models for TCS and signal transduction PR. In addition we also search the primary literature for known circuits that have been characterized biochemically and collected all different parameter values for the various reactions in the PR circuit. This information is compiled in Table S2, revealing that kinetic parameters for corresponding reactions in experimentally well-characterized systems are quite similar and, in most cases, within the same order of magnitude.

### Calculating the dynamic behavior of each model

#### Steady state concentrations and stability

We obtained steady state concentrations by numerically solving the models for each set of parameter values. We then calculated the Jacobian of the ODE system and the eigenvalues of that Jacobian to determine the stability of the steady state. We compared the steady state stability of the PR circuits in two ways. As we selected for sets of parameter values that generate systems with stable steady states (see methods), all real parts of the eigenvalues of the steady state are negative. Taking this into account we compared the minimum of the real parts of the eigenvalues in each pair of models. This comparison allows us to compare the fastest time scale in which the two circuits respond to a transient perturbation to their steady states. We also compared the maximum of the real parts of the eigenvalues between the two circuits in each pair. This allows us to compare the slowest time scale in which the two circuits respond to a transient perturbation to their steady states.

#### Logarithmic gains and parameter sensitivities

To estimate the response of a physiological variable to changes in environmental signals or model parameters, we compute (Alves et al., 2008)

\begin{eqnarray*}L \left( X,\theta \right) = \frac{dLog(X)}{dLog(\theta )} ={ \left( \frac{\theta }{X} \times \frac{dX}{d\theta } \right) }_{SS} \end{eqnarray*}

Here, ss indicates evaluation at a reference steady state.

#### Signal amplification

To estimate signal amplification we calculated the logarithmic gains of the concentration of the phosphorylated form of the final response regulator (RR2) with respect to the cognate signal of the system (Signal), as given by:

\begin{eqnarray*}L \left( RR2P,Signal \right) = \frac{dLog(RR2P)}{dLog(Signal)} = \frac{Signal}{RR2P} \frac{dRR2P}{dSignal} \end{eqnarray*}

In our case the Signals are the rate parameters for the self-phosphorylation and self-dephosphorylation reactions of the SK.

#### Sensitivity to the total amount of protein

To estimate sensitivity of the different phosphorylated forms of the PR proteins (Prot-P) with respect to the total amount of circuit proteins (Pr_tot_), we calculated the logarithmic sensitivities, as given by:

\begin{eqnarray*}L \left( Prot-P,P{r}_{tot} \right) = \frac{dLog(Prot-P)}{dLog(P{r}_{tot})} = \frac{P{r}_{tot}}{Prot-P} \frac{dProt-P}{dP{r}_{tot}} \end{eqnarray*}

#### Global sensitivity to fluctuations in parameters

To estimate sensitivity of the different phosphorylated forms of the PR proteins (Prot-P) with respect to fluctuations in each of the parameters (Par _i_), we calculated the logarithmic sensitivities, as given by:

\begin{eqnarray*}S \left( Prot-P,Pa{r}_{i} \right) = \frac{dLog(Prot-P)}{dLog(Pa{r}_{i})} = \frac{Pa{r}_{i}}{Prot-P} \frac{dProt-P}{dPa{r}_{i}} \end{eqnarray*}

Then, for each protein, we calculate the norm of the vector whose coordinates are the individual sensitivities of that protein, as described in (Savageau, 1971a; Kitano, 2002). Thus, for architecture *Mi*, the aggregated sensitivity is given by

\begin{eqnarray*}{S}_{aggregate,Mi}=\sqrt{\sum _{i=1}^{n}S(Prot-P,Pa{r}_{i})^{2}} \end{eqnarray*}

This is an aggregated measure of the robustness of circuits to fluctuations in parameter values (Alves et al., 2008).

#### Response times

We performed two independent experiments per architecture and per set of parameter values to calculate the response times. First, we start with the system fully dephosphorylated and run a time course simulation using the same scan procedure for the self-phosphorylation and self-dephosphorylation rate constant of SK. We then calculate the time it takes each of the architectures to reach 90% of the new steady state values. We repeated this experiment starting with fully phosphorylated proteins.

#### Amount of information transmitted through the circuit

To calculate the information transmitted through the PR we simulated that system to steady state using Gillespie’s algorithm for stochastic simulation one hundred times per set of parameter values (Gillespie, Hellander & Petzold, 2013). The proxy for changes in the environmental information was taken to be the number of phosphorylated molecules of the SK domain, as this is the sensing domain of the PR. The output of the PR was considered to be the number of phosphorylated final response regulators. A high correlation between the variations in the amount of phosphorylated SK and RR2 implies that information is transmitted in higher amounts through the circuit.

To calculate the amount of information being transmitted through the circuit we started by running twenty five stochastic simulations with each set of parameter values and initial conditions. At steady state, we sampled each simulation at one second intervals, running the steady state for 20 min. We pooled together the simulations and calculated the individual relative frequencies, for the number of SKP, RR1P, HptP and RR2P molecules. We also calculated the paired relative frequencies for (SKP, RR2P). We then used these numbers to estimate the information being transmitted through the circuit by applying the standard formula for mutual information between the two variables (Bekker, Teixeira de Mattos & Hellingwerf, 2006; Lan & Tu, 2016):

\begin{eqnarray*}MI \left( SKP\rightarrow RR2P \right) =\sum _{SK{P}_{i}}\sum _{RR2{P}_{j}}p(SKPi,RR2Pj)\log \nolimits ( \frac{p(SKPi,RR2Pj)}{p(SKPi)p(RR2Pj)} ) \end{eqnarray*}

In these formulas, *p*(*x*) is the relative frequency of *x*, and *p*(*x*, *y*) is the joint relative frequency of *x* and *y*. $MI \left( SKP\rightarrow RR2P \right) $ estimates the amount of information being transmitted through the circuit.

We calculated $MI \left( SKP\rightarrow RR2P \right) $ as a simultaneous function of the rate constants for the phosphorylation and dephosphorylation reactions of the SK and RR2. To present this information in a way that is visually easier to interpret, we calculate an aggregated mutual information defined as:

\begin{eqnarray*}M{I}_{agregated} \left( SKP\rightarrow RR2P \right) =\sum _{k=kmin}^{kmax}MI \left( SKP\rightarrow RR2P \right) \end{eqnarray*}

Here, *k* represents the rate constant that is not explicitly represented in the plots.

### Comparison of dynamic properties between architectures

#### Numerical comparison of mechanistic models

We took a reference architecture and generated a latin hypercube sampling for the parameter values of the mechanistic models of architecture M1. We approximated the experimentally determined protein abundances to the order of magnitude and then also generated a latin hypercube sampling strategy, which we combined with that for the parameter values. Overall, we performed several hundred million sets of simulations.

#### Mathematical modelling of the Spo0 and Sln1 phosphorelays

Detailed mechanistic models were created for the Spo0 and Sln1 phosphorelays, as described in Appendix S1. These models use experimental data from Narula et al. (2015) for Spo0 and from the SGD (Hirschman, 2006) for Sln1.

Then, for each of the PR, four additional equivalent models were created, each assuming that the PR would have a different architecture. The parameter values for these alternative architectures were considered to be the same as those for the native circuit architecture. The concentrations for the proteins were optimized by first simulating the original model at different signal intensities and calculating the steady state phosphorylation state of the final RR. Using these results we then performed the same experiments for each alternative architecture and allowed for the proteins that got fused or separated with respect to the cognate architecture to change in concentration by up to one order of magnitude below or above that in the natural architecture. We then selected the concentrations that led to the most similar signal-response curves using a least square minimum criteria for the differences between phosphorylated final RR.

#### Calculating the cost of alternative architectures for the Spo0 and Sln1 phosphorelays

To calculate the cost of alternative architectures for the Spo0 and Sln1 we first counted the number of amino acids in the sequence for each protein in the original architecture. Then, we assumed that any alternative architecture would result from fusing or splitting the original proteins while conserving the same number of amino acids. Finally, we multiplied the number of amino acids of the individual proteins in the phosphorelay by the number of proteins in the cell and used this number as a proxy for the cost of each architecture. This makes it so that the cost of maintaining one copy of the PR circuit is the same between the alternative architectures. Thus, differences in the metabolic cost for alternative architectures of the Spo0 (or Sln1) PR circuit are simplified to the differences in ATP consumption rate. As we also adjusted concentrations of each alternative architecture to make the signal response curves of the alternative architectures as similar as possible to that of the native architecture (see below), this makes differences in the cost of circuit maintenance equate to differences in protein concentration.

#### Software

All models and analysis were done using Mathematica (Wolfram Research I, 2014). The notebooks containing all code to generate each figure are given as supplementary data pack S1.

## Results

### Physiological variables as a proxy for circuit performance in signal transduction

The overall fitness of organisms is affected by how their molecular components organize into biological circuits (Savageau, 1976; Alon, 2007). The organization of molecular circuits with common biological functions in different organisms may have alternative architectures. Often, these alternatives lead to improved circuit performance in the context of the organisms where they are observed. Understanding how the alternative architectures affect circuit performance requires that the physiological variables that are important for the biological function of the circuit are understood. Over the last few decades, several of these physiological variables were identified as important in determining the performance of molecular signal transduction circuits. We compiled those variables and summarize them in Table 1.

### What physiological variables are optimized by native architectures in kinetically well characterized PRs?

To answer this question, we focus on two systems for which abundant quantitative and kinetic information is available: The Spo0 PR in *Bacillus subtilis* and the Sln1 system in *Saccharomyces cerevisiae*.

### The Spo0 phosphorelay of *Bacillus subtilis*

**Figure 2:**
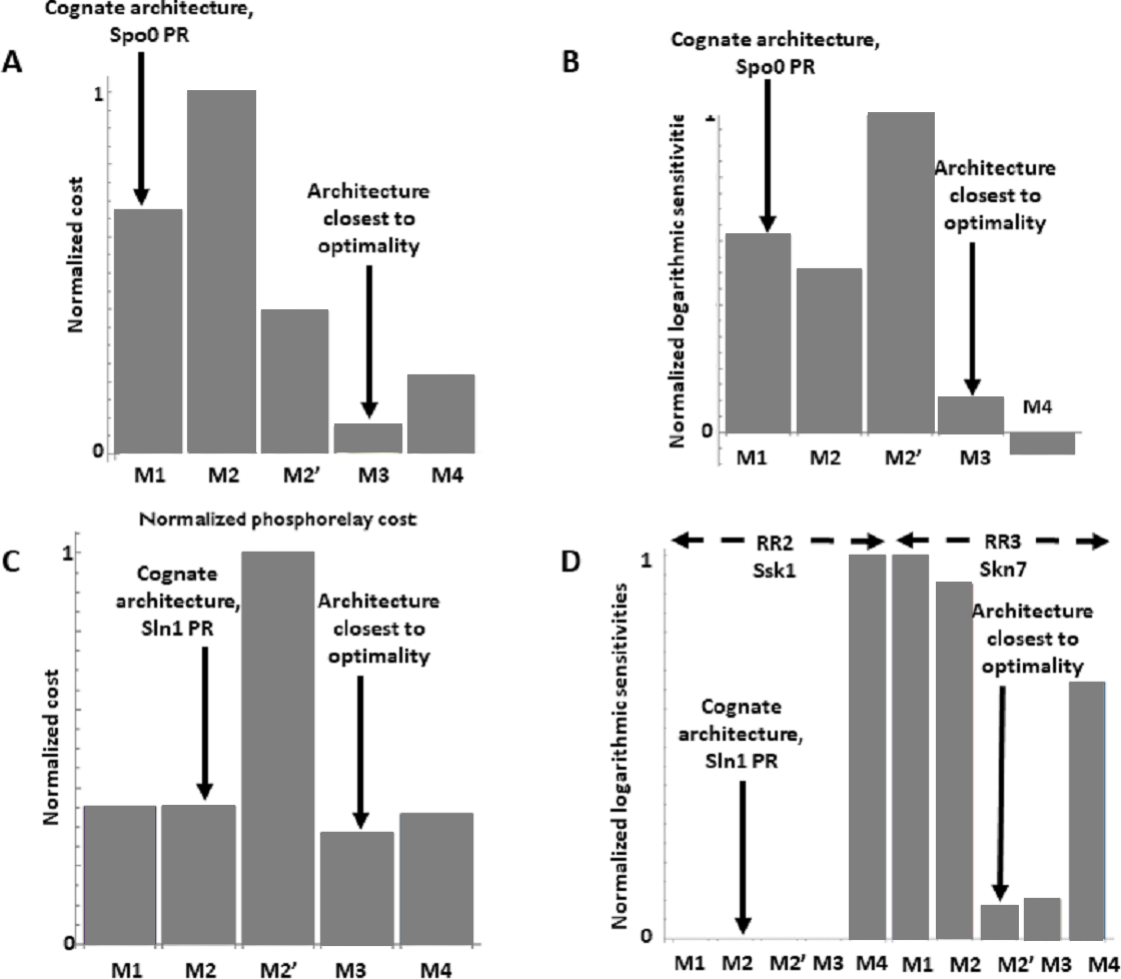
Effects of architectures on metabolic cost and robustness of the response for the Spo0 phosphorelay of *Bacillus subtilis* and the Sln1 phosphorelay of *Saccharomyces cerevisiae*. In all cases, the protein amounts of the alternative architectures were optimized to make the steady state signal-response curves be as similar as possible (see methods). A and B to the Spo0 phosphorelay. C and D pertain to the Sln1 phosphorelay. (A, C) Cost of synthesizing the circuit under different architectures. X–axis: PR architecture. Y–axis: total metabolic cost of the circuit proteins (arbitrary units). (B, D) Normalized sensitivity of the steady state concentration of the final response regulator to changes in parameters. X–axis: PR architecture. Y–axis: euclidean norm of the sensitivities vector of the response regulator.

**Figure 3:**
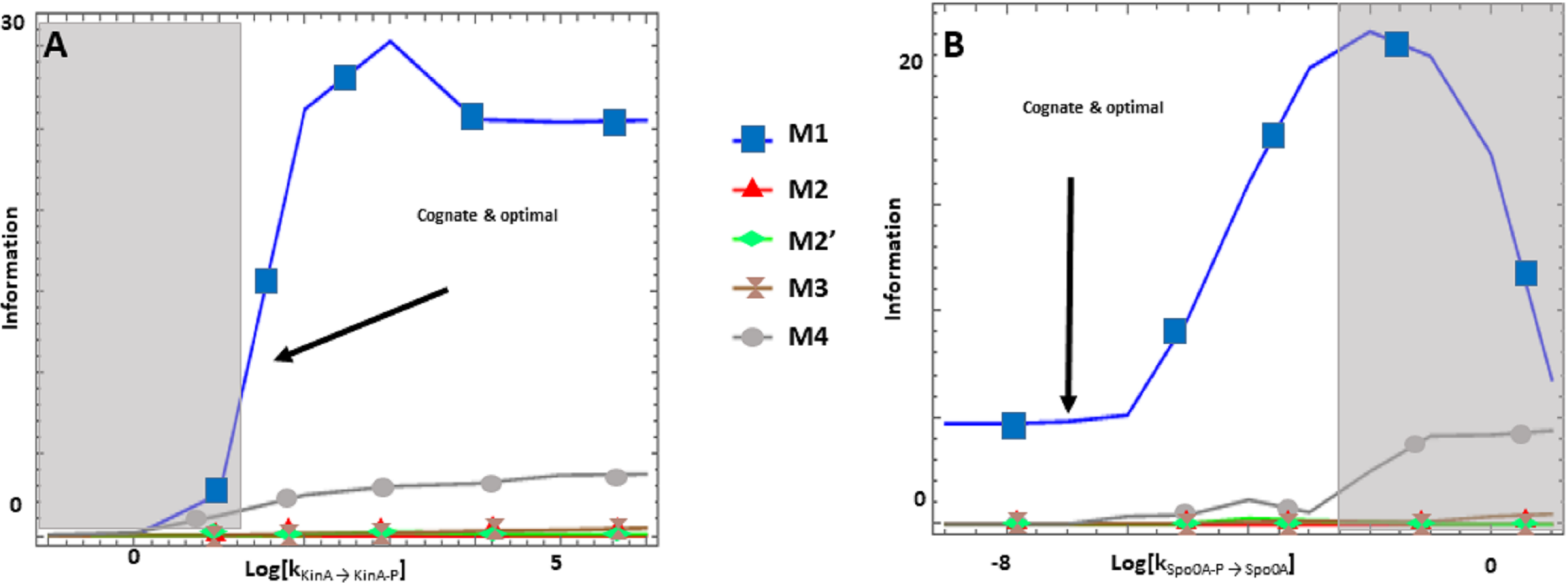
(A-B) Effects of alternative architectures in the transmission of information through the Spo0 phosphorelay of *Bacillus subtilis*. In all cases, the protein amounts of the alternative architectures were optimized to make the steady state signal-response curves be as similar as possible. The *Y*-axis represents the accumulated mutual information over a range of six orders of magnitude for the self-dephosphorylation rate constant of kinA between variations in the number of phosphorylated kinA molecules and that of phosphorylated Spo0E molecules. k_KinA→KinA−P_ represents modulation of the kinA phosphorylation rate, while k_Spo0A→Spo0A−P_ represents modulation of SpoA dephosphorylation. Architecture M1 transmits the most information for comparable parameter values.

The Spo0 phosphorelay in *B. subtilis* is probably the PR system with the most abundant and reliable experimental determinations of kinetic information (Grimshaw et al., 1998; Eswaramoorthy et al., 2010; Sen, Garcia-Ojalvo & Elowitz, 2011; Narula et al., 2012; Narula et al., 2015). The architecture of this system is of type M1, and Appendix S1 gives the protein abundances and parameter values for the reactions. We created a mass action mathematical model describing the dynamic behavior of the native M1 architecture for this PR. We then created mathematical models for the four alternative PR architectures (M2–M4) with the same parameter values. Protein abundances were adjusted in each case to minimize differences in the signal response curve with respect to the native circuit architecture. Then, we compared the architectures with respect to the variables in Table 1. We found that the cost of protein synthesis is an order of magnitude lower for architecture M3 than for M1 (Fig. 2A). Protein costs for architectures M1, M2′, and M4 are an order of magnitude higher than in M3 and one order of magnitude lower than in M2. The robustness of signal transmission to environmental fluctuations in parameters is smaller in architectures M1 and M2′ and larger in M2, M3, and M4, as shown by the higher aggregate sensitivities of the latter architectures with respect to the former ones (Fig. 2B). Architecture M4 is the fastest in responding to phosphorylating signals, and M4 and M1 are faster in a similar percentage of cases when the signal dephosphorylates the system (Table 2). Thus, M4 is statistically optimal with respect to fast adaptation. M1 transmits the highest amounts of information about the environment over the regulatable signal range for the Spo0 phosphorelay, whether the signal comes at the level of the SK (modulation of k1) or at the level of regulating dephosphorylation of the final response regulator (modulation of k18, Fig. 3). Taken together, these results suggest that the native architecture of the Spo0 phosphorelay (M1) optimizes the amount of information transmitted to the cell about changes in the environmental signal over the regulatable signal range. The other analyzed performance goals seem to be of secondary importance.

**Table 2:** Percentage of simulations where each architecture was fastest to reach new steady state for the Spo0 and Sln1 PR.

	***Spo0 System***	
Architecture	Type of signal	
	Inducing Phosphorylation 8000 simulations	Inducing Dephosphorylation 8000 simulations
M1 (Cognate)	0.5	34.3
M2	6.7	18.3
M2′	2.7	6.1
M3	4.4	4.6
M4 (optimal)	85.7	36.6

	***Sln1 System***	
Architecture	Type of signal	
	Inducing Phosphorylation 8000 simulations	Inducing Dephosphorylation 8000 simulations
M1	0	100
M2 (Cognate)	30.9	0
M2′	5.3	0
M3	10.5	0
M4 (Optimal)	53.3	0

### The Sln1/Ypd1/Ssk1 and Sln1/Ypd1/Skn7 phosphorelays of *Saccharomyces cerevisiae*

*S. cerevisiae* uses a PR with architecture M2 to sense changes in the osmolarity of the medium and regulate its internal metabolism and membrane composition, adapting to those changes. The hybrid sensor kinase Sln1 senses changes in membrane curvature and ultimately modulates the phosphorylation state of the terminal response regulators Ssk1 and Skn7. Phosphorylation of Ssk1 acts as a molecular switch in controlling the yeast’s osmosensing mitogen-activated protein (MAP) kinase cascade (Janiak-Spens, Cook & West, 2005), while phosphorylation of Skn7 directly affects the transcription of SLN1-SKN7-responsive genes (Fassler & West, 2013). Skn7 is also involved in regulating heat shock response in a Sln1-Ypd1 independent way (Fassler & West, 2013). Reliable kinetic parameter values and protein abundances are available for this PR.

We use those values to create a PR model where we consider the simultaneous presence of the terminal response regulators Ssk1 and Skn7. Then, create four independent alternative models, with architectures M1, M2′, M3, and M4 and parameter values equal to those of the native circuit architecture. Finally, we optimize protein abundance to minimize differences in the dynamic signal-response curve of each alternative architecture with respect to that of the native architecture of the system.

**Figure 4:**
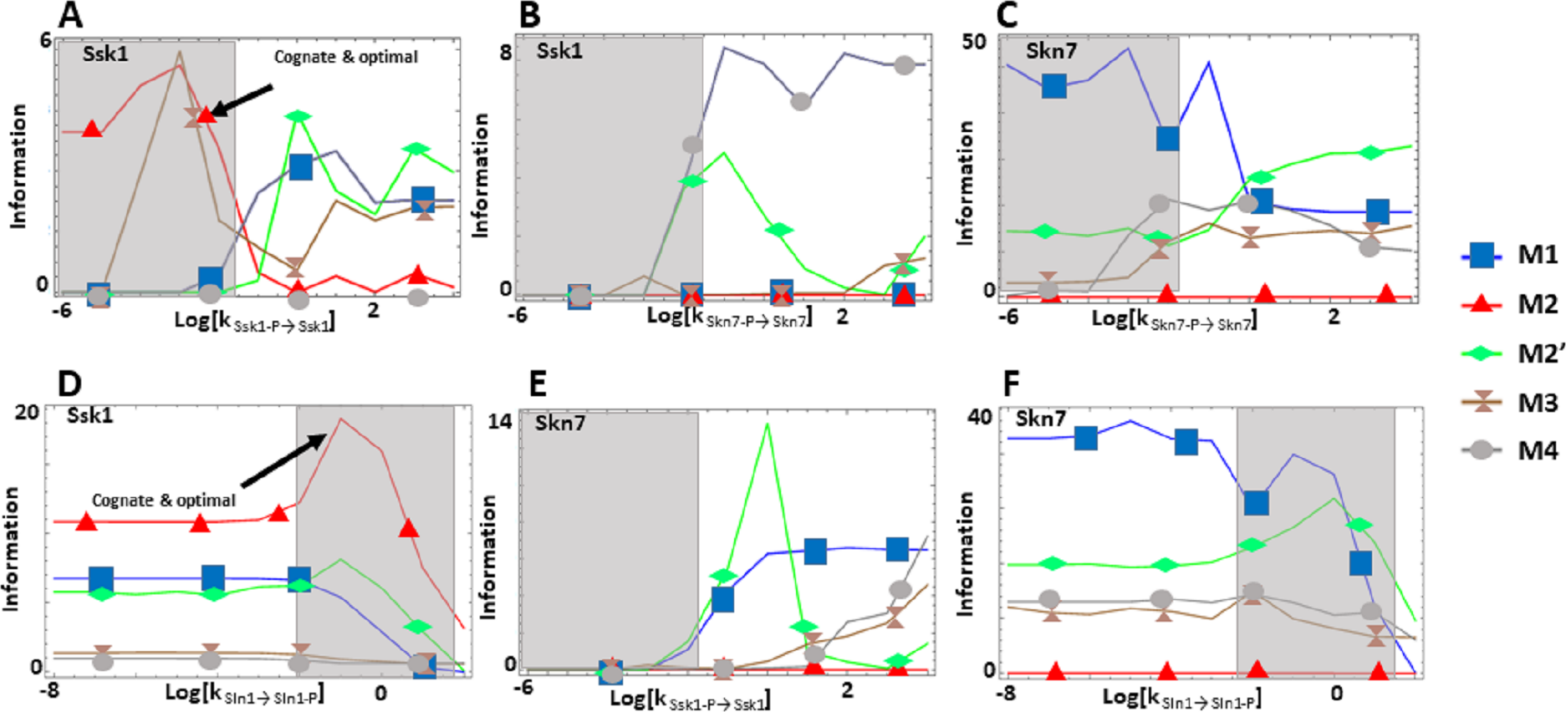
(A-F) Effects of alternative architectures in the transmission of information through the Sln1 phosphorelay of *Saccharomyces cerevisiae*. In all cases, the protein amounts of the alternative architectures were optimized to make the steady state signal-response curves be as similar as possible. The *Y*-axis represents the accumulated mutual information over a range of six orders of magnitude for the self-dephosphorylation rate constant of Sln1 between variations in the number of phosphorylated Sln1 molecules and that of phosphorylated Ssk1 or Skn7 molecules. k_Sln1→Sln1−P_ represents modulation of the Sln1 phosphorylation rate, k_Ssk1-P→Ssk1_ represents modulation of Ssk1 dephosphorylation, and k_Skn7-P→Skn7_ represents modulation of Skn7 dephosphorylation. Architecture M2 transmits the most information to Ssk1 for comparable parameter values.

We find that the cost of architecture M2′ is one order of magnitude higher than that of each of the other architectures, which have a similar metabolic cost (Fig. 2C). The sensitivity of the steady state concentrations for the Ssk1 RR are low and similar in all alternative architectures except M4, where sensitivity is high. In contrast, the sensitivity of Skn7 concentration is highest in architectures M1 and M2 (Fig. 2D). Architecture M4 most frequently responds faster to signals that increase the phosphorylation levels of the proteins, followed by M2 (Table 2). In contrast, architecture M1 is always the fastest if the change in environmental conditions decreases phosphorylation levels of the circuit. When it comes to transmitting information about the environment to Skk1 over the regulatable range of the circuit, architecture M2 is the best for a wider range of parameters. It surprised us that all non-native architectures are better information transmitters to Skn7 than M2 (Fig. 4; see discussion for a rationale on why this may be so). It is interesting to note that only very high rates of dephosphorylation for one RR affect the information transmitted by the circuit to the other, increasing it significantly.

Overall, the analysis of these two systems suggests that transmission of information is an important determinant of architectural selection in PR. While different architectural solutions are used in each case (M1 for Spo0 and M2 for Sln1), the native circuit architectures maximize information transmission for the parameter values and typical concentration ranges of each system.

### Analyzing real PR examples: How do different PR architectures influence information transmission?

#### A performance atlas for information transmission in the parameter space of PR architectures

With the hypothesis that information transmission is important in the selection of PR architecture, we created a data-driven atlas that describes how architecture, protein abundance, and parameter values influence information transmission in PRs. To create that atlas we compiled experimentally determined parameter values and proteins abundances for as many PR systems as we could find in the primary literature and in public databases (see methods and Appendix S1). Then we used those parameters and protein abundances to calculate the capacity to transmit information of each alternative architecture with all possible combinations of protein abundances and kinetic values. Finally we ranked the five architectures with respect to increasing capacity of information transmission. The atlas is summarized in Table S3.

#### Experimentally determined protein abundances are consistent with amount of transmitted information being an important driver of PR architecture selection

We identified and obtained protein abundances for seventeen PR systems that are present in the PAXDB database (Wang et al., 2015) and belong to seven different organisms (Table S1). Then we matched these abundances with the performance in the atlas of amount of transmitted information as a function of parameter values, protein abundance and circuit architecture given in Table S3. In 16 out of the 17 cases we find that the native architecture of the PR systems is consistent with maximizing the through-circuit transmitted information under experimentally known parameter conditions (Table 3).

**Table 3:** Observed native architectures and predictions of where in parameter space their observation is expected.

Organism	Phosphorelay	Ratios of abundance (order of magnitude)	Estimated proteins per cell ^h^	Predicted operational regions
*Escherichia coli*	TorS:TorR (M3)	1:1:1:10	100:1000	***k1, k2>10***^−1^***s***^−1^^a^
	EvgS:EvgA (M3)	1:1:1:100	100:10000	***k1, k2>10***^−1^***s***^−1^^a^
	BarA:UvrY (M3)	1:1:1:10	1000:10000	***k1, k2>10***^−1^***s***^−1^^a^
	ArcB:ArcA (M3)	1:1:1:10	10000:1000000	***k1, k2>10***^−1^***s***^−1^^a^
	RcsC:RcsD:RcsB (M2)	1:1:1:100	1000:1000:100000	***k1>10***^−1^***s***^−1^^b^
*Shigella flexneri*	BarA:UvrY (M3)	1:1:1:1	1000:1000	M1 or M2′
	ArcB:ArcA (M3)	1:1:1:10	10000: 100000	***k1>10***^−1^***s***^−1^^c^
*Shewanella oneidensis*				
	SO0859:SO0860 (M3)	1:1:1:1	100000:100000	***k1>10***^−1^***s***^−1^^d^
*Desulfovibrio vulgaris*	DVU_3062:DVU_3061 (M3)	1:1:1:1	100000:100000	***k1>10***^−1^***s***^−1^^d^
				
*Saccharomyces cerevisiae*	Sln1:Ypd1:Ssk1 (M2)	1:1:1:1	1000:1000:1000	^e^
	Sln1:Ypd1:Skn7 (M2)	1:1:1:1	1000:1000:1000	^e^
				
*Schizosaccharomyces pombe*	Mak1:Mpr1:Mcs4 (M2)	1:1:1:10	1000:1000:10000	^f^
	Mak2:Mpr1:Mcs4 (M2)	1:1:1:10	1000:1000:10000	^f^
	Mak3:Mpr1:Mcs4 (M2)	1:1:1:10	1000:1000:10000	^f^
*Bacillus subtilis*	KinA:Spo0F:Spo0B:Spo0A (M1)	1:100:1:100	12:4200:110:1700	***k1, k2<10***^−1^***s***^−1^***.***^g^
	KinB:Spo0F:Spo0B:Spo0A (M1)	1:100:1:100	93:4200:110:1700	***k1, k2<10***^−1^***s***^−1^***.***^g^
	KinC:Spo0F:Spo0B:Spo0A (M1)	1:100:1:100	82:4200:110:1700	***k1, k2<10***^−1^***s***^−1^***.***^g^

**Notes.**

a As compared to architectures M1, M2, M2′. M4 does not allow for the observed abundance ratio between signal transduction domains.

b As compared to M1, the only other architecture that allows for the observed ratio between abundances of signal transduction domains. Regulation by the environment expected at the SK phosphorylation step.

c As compared to architectures M1, M2, M2′. M4 does not allow for the observed abundance ratio between signal transduction domains. Regulation by the environment expected at the SK phosphorylation step.

d As compared to architectures M1, M2, M2′ and M4. Regulation by the environment expected at the SK phosphorylation step.

e See analysis of the system as a Sln1-Ypd1-Skn7-Ssk1 PR in the main text.

f Outside the range of protein abundances tested in this work. Nevertheless, comparing the trends of similar abundance ratios for one order of magnitude less suggests that M2 would be the preferred architecture if we pool the abundances of Mac1, Mac2 and Mac 3 proteins together.

g Comparison between architectures M1 and M2′, which are the only ones that allow for this ratio of abundance between domains. Consistent with experimental determinations of the rate constants (supplementary materials).

h These numbers are calculated by multiplying cell volume (µm^3^), average number of proteins in cells per µm^3^, and the protein abundance in parts per million: *CellVolume* × 6.23 × 10^8^ × *Proteinabundances* × 10^−6^.

## Discussion

### Implications

Mutations in the genomes of organisms led to the emergence of variant architectures for biological circuits with similar biological function. Natural selection acting upon this variability led to fixing those architectures that are more efficient for the function of the circuit in the genome, contingent on life history. Elucidating the reasons why these architectures improve performance of the circuit reveals biological design principles that constrain evolution and explain the observed patterns.

In some cases, biological design principles are general and apply to a whole class of biological circuits. For example, overall feedback inhibition of the final product to the initial reaction of a linear biosynthetic pathway leads to molecular systems that are faster, more responsive, and less sensitive to fluctuations than any other regulatory alternatives (Alves & Savageau, 2000a; Alves & Savageau, 2000b; Alves & Savageau, 2001). As such that regulatory solution is widespread in the tree of life. However, in many cases, design principles appear casuistic and are specific to a single circuit. For example, the circuit that regulates competence in *B. subtilis* appears to have been selected over alternative designs because of its noisy response (Cağatay et al., 2009), which enables bet edging in the competence response of the bacterium and improves its survival chances.

In the case of PR, a specific architecture is observed in over 72% of all genomic PR (Salvado et al., 2015) in a sample of more than 9,000 organisms (Salvado et al., 2015). This suggested that indeed there could be design principles for selecting the architecture of these circuits. To test this hypothesis we needed to understand what physiological variables influence the performance of the circuit. Then, we needed to study how architecture affects those variables, allowing natural selection between alternative PR architectures.

Initially we compiled a list of physiological variables that are important for the function of these signal transduction circuits (Savageau, 1971b; Savageau, 1971a; Alves & Savageau, 2003; Igoshin, Alves & Savageau, 2008; Shinar & Feinberg, 2010; Shinar & Feinberg, 2011; Shinar & Feinberg, 2013; Csikász-Nagy, Cardelli & Soyer, 2011; Sen, Garcia-Ojalvo & Elowitz, 2011; Ray, Tabor & Igoshin, 2011; Salvado et al., 2012; Knudsen, Feliu & Wiuf, 2012; Kothamachu et al., 2013; Amin et al., 2014; Uschner & Klipp, 2014; Feng et al., 2016). We found that information transmission is maximized by the native architectures of kinetically well characterized PR Spo0 of *B. subtilis* and Sln1 of *S. cerevisiae*. This result led us to hypothesize that optimization of information transmission is an important driver of selection for PR architectures. This is consistent with previous studies of TCS suggesting that selection to maintain efficient signal transmission through a circuit appears to be sufficient to prevent loss of specificity in the interactions between the various signal transduction domains of the circuit (Rowland & Deeds, 2014). Those results indicate that, after genomic duplication of a TCS, the signal sensing domain of the histidine kinase evolves at a slower rate than that of the interface between sensor kinase and the response regulator. This differential evolution keeps specificity of the circuits and minimizes crosstalk (46–49).

To investigate this hypothesis in a data driven manner, we created an atlas of how architecture, protein abundance, and parameter values influence information transmission capacity. To create that atlas we collected experimentally determined parameter values and proteins abundances for as many PR systems as we could find in the primary literature and in public databases. Then we used those parameters and protein abundances to calculate the capacity to transmit information of each alternative architecture with all possible combinations of protein abundances and kinetic values. Finally, we selected seventeen PR from different organisms and mapped them onto the performance atlas by using their experimentally determined protein abundances. The native architecture of the system is the one that maximizes information transmission in sixteen out of seventeen cases.

Overall, the results of our analysis for well characterized PR circuits are consistent with the hypothesis that information transmission is a crucial driver of PR circuit selection in nature. This was previously suggested to be the case for MAP kinase signaling and other metabolic circuits in eukaryotes (Paulsson & Ehrenberg, 2000; Paulsson, Berg & Ehrenberg, 2000; Pedraza & Paulsson, 2007; Pedraza & Paulsson, 2008; Lestas, Vinnicombe & Paulsson, 2010; Hilfinger & Paulsson, 2011; Cheong et al., 2011; Nemenman, 2013).

Still, architecture selection is a complex function of the interplay between the architecture itself, protein amounts and kinetic parameters, and evolution can play with these “genotypic variables” to find instances of each architectures that are almost equivalent information transmission circuits. Although we did look for additional specific examples of PR, we could not find any for which a complete set of parameter values had been measured either by the same or by independent labs. For some of them there were partial sets of parameter values (The ArcA –ArcB, the EvgS –EvgA, and the BvgS –BVgA PR). Nevertheless, creating models for these systems would have required using more than half of the parameters from other systems. This would have created a situation where the model would not be driven from the data, but rather from other estimates.

Our results suggest that maximization of information could be a design principle that is consistent with the evolutionary selection of PR architectures. These results also have consequences for synthetic biology. If we want to build synthetic PR that are stable in the genome, and have a significant level of quantitative understanding about the operating ranges for the circuit, then it should be designed using the architecture that has the highest capacity to transmit information over that range.

### Speculations

Our analysis invites some speculation regarding the ranges of operability for many PR. If 72% of all PR have a M3 architecture, as a first hypothesis we could expect that these systems operate in the ranges where the M3 architecture outperforms the others. By looking at our performance in information transmission atlas, this implies that M3 architecture systems are likely to be operating at abundance ranges of the PR that are below 100 molecules per cell for the protein containing the SK domain. This is amenable to testing in future proteomic experiments. The M3 circuits are also expected to be operating over modulatable ranges of the self-phosphorylation and self-dephosphorylation rate constants of SK above 10^−1^ min^−1^. Similarly, 18% of the identified PR might be operating mostly over the range where architecture M2′ transmits a high amount of information about the system, and 6% over the range where M4 is a better information transmitter.

In addition, we speculate that the type of environmental stimulus is also important in selecting the architecture. If the stimulus changes in a graded way and cells can be adjusted in a similar graded way, it makes sense that the architecture should allow for a high capacity transmission. For example, an architecture that allows the cell to distinguish between n+1 states (that is, with a capacity to transmit information of n+1 bits) provides for a better design that another architecture that only distinguishes between n states. On the other hand, if a very sharp response is required, architectures that can distinguish between a small number of states over a short operability range might be more effective.

Another tempting speculation arises from our modelling of the Sln1 phosphorelay. Given its information transmission profile, it could be that Sln1-mediated activation of Skn7 and its dependent genes may not be an important function of the circuit. For the experimentally determined parameter values and protein concentrations, other architectures would transmit more information to that RR. This suggests to us that Skn7’s role in heat shock response might be much more important for the cell than its role in osmoregulation.

### Limitations

Our study has several limitations. Here we discuss those we think are the most important. First, the performance landscape of PR can be strongly affected by how its expression is regulated (Cağatay et al., 2009; Narula et al., 2010; Ray, Tabor & Igoshin, 2011; Tiwari & Igoshin, 2012). In fact Ray & Igoshin (2012) have shown that noise in the circuit is likely to be dominated by gene expression, rather than protein fluctuations, which is the level we analyze. Nevertheless, transcriptional regulation occurs on a timescale of tens to hundreds of minutes, while the regulation of phosphorylation levels of the PR occurs on a timescale of minutes and this timescale can also influence the performance of the PR signal transduction circuit (Tiwari et al., 2010; Tiwari & Igoshin, 2012).

Second there might be a wider range of parameter values for each reaction of the PR in the wild and this could change our phenotypical mapping of PR behavior onto the phase and architectural spaces of the circuits. Nevertheless, if this is so, our results would still be valid for the regions that were analyzed and considering that expanded set of values would only increase the “genotypic space” without changing the mapping we present here.

Third, our analysis of the capacity to transmit information focuses on steady state shifts. One could argue that the transient capacity could be a more important determinant of system performance. However, we tested how the transient capacity to transmit information compares to the corresponding steady state capacity and found that the latter is always an upper limit of the former.

Fourth and final, our initial comparative analysis is based on two examples for which there is quantitative information for the complete set of kinetic parameters and protein abundances. All other PR systems we looked at lacked information about parameter values, protein abundances or both. Still, when we identify 17 PR systems for which there is a complete set of experimentally determined protein abundances, their cognate architecture is the one we would expect if information transmission was being optimized by evolution, with one exception.

##  Supplemental Information

10.7717/peerj.11558/supp-1 Supplemental Information 1 Mathematical modelling This file presents the mathematical models, kinetic parameter values and proteins numbers used for the simulations.

10.7717/peerj.11558/supp-2 Supplemental Information 2 Phosphorelay circuits for which there is experimental abundance information

10.7717/peerj.11558/supp-3 Supplemental Information 3 Numerical values of the rate constants of the reactions in the PR models used in the stochastic simulations, represented in Fig. 4

10.7717/peerj.11558/supp-4 Supplemental Information 4 Atlas for the behavior of the various architectures under varying parameters, ratios between signal transmission domains, and absolute proteins abundances

10.7717/peerj.11558/supp-5 Supplemental Information 5 Mathematica notebook providing the code for the simulations and figures

## Additional Information and Declarations

### Data Availability

The following information was supplied regarding data availability: The Mathematica notebooks used to generate all simulations and figures are available as a Supplementary File.
