# Supplementary material for: Maximization of information transmission influences selection of native phosphorelay architectures
Source: PeerJ. 2021 Jun 10;9:e11558. doi: 10.7717/peerj.11558 (PMC8199921; doi:10.7717/peerj.11558)
Supplement: Supplemental Information 1 — This file presents the mathematical models, kinetic parameter values and proteins numbers used for the simulations. [file peerj-09-11558-s001.docx]

**What influences selection of native phosphorelay architectures?**

# Supplementary text S1 – Mathematical modelling

## Conceptual schemes and mass action mathematical models

### Architecture M1

| Reaction | Rate |
| --- | --- |
| SK → SKP | k_1_ × SK |
| SKP → SK | k_2_ × SKP |
| SKP + RR1 → SKPRR1 | k_3_ × SKP × RR1 |
| SKPRR1 → SKP + RR1 | k_4_ × SKPRR1 |
| SKPRR1 → SKRR1P | k_5_ × SKPRR1 |
| SKRR1P → SK + RR1P | k_6_ × SKRR1P |
| SK + RR1P → SKRR1P | k_7_ × SK × RR1P |
| SKPRR1 → SKRR1 | k_8_ × SKPRR1 |
| SKRR1 → SK + RR1 | k_9_ × SKRR1 |
| SK + RR1 → SKRR1 | k_10_ × SK × RR1 |
| RR1P → RR1 | K_11_ × RR1P |
| RR1P + Hpt →RR1PHpt | k_12_ × Hpt × RR1P |
| RR1PHpt → RR1P+Hpt | k_13_ × RR1PHpt |
| RR1PHpt → RR1HptP | k_14_ × RR1PHpt |
| RR1HptP → RR1PHpt | k_24_ × RR1HptP |
| RR1HptP → RR1 + HptP | k_15_ × RR1HptP |
| RR1 + HptP → RR1HptP | k_16_ × RR1 × HptP |
| HptP → Hpt | k_17_ × HptP |
| HptP + RR2 → HptPRR2 | k_18_ × HptP × RR2 |
| HptPRR2 → HptP + RR2 | k_19_ × HptPRR2 |
| HptPRR2 → HptRR2P | k_20_ × HptPRR2 |
| HptRR2P → HptPRR2 | k_25_ × HptRR2P |
| HptRR2P → Hpt + RR2P | k_21_ × HptRR2P |
| Hpt + RR2P → HptRR2P | k_22_ × Hpt×RR2P |
| RR2P → RR2 | k_23_ × RR2P |

### Architecture M2

| Reaction | Rate |
| --- | --- |
| SKRR1 → SKPRR1 | k_1_ × SKRR1 |
| SKPRR1 → SKRR1 | k_2_ × SKPRR1 |
| SKPRR1 → SKRR1P | k_5_ × SKPRR1 |
| SKPRR1 → SKRR1 | k_8_ × SKPRR1 |
| SKRR1P → SKRR1 | K_11_ × SKRR1P |
| SKRR1P + Hpt →SKRR1PHpt | k_12_ × Hpt × SKRR1P |
| SKRR1PHpt → SKRR1P+Hpt | k_13_ × SKRR1PHpt |
| SKRR1PHpt → SKRR1HptP | k_14_ × SKRR1PHpt |
| SKRR1HptP → SKRR1PHpt | K_24_ × SKRR1HptP |
| SKRR1HptP → SKRR1 + HptP | k_15_ × SKRR1HptP |
| SKRR1 + HptP → SKRR1HptP | k_16_ × SKRR1 × HptP |
| HptP → Hpt | k_17_ × HptP |
| HptP + RR2 → HptPRR2 | k_18_ × HptP × RR2 |
| HptPRR2 → HptP + RR2 | k_19_ × HptPRR2 |
| HptPRR2 → HptRR2P | k_20_ × HptPRR2 |
| HptRR2P → HptPRR2 | k_25_ × HptRR2P |
| HptRR2P → Hpt + RR2P | k_21_ × HptRR2P |
| Hpt + RR2P → HptRR2P | k_22_ × Hpt×RR2P |
| RR2P → RR2 | k_23_ × RR2P |

### Architecture M2’

| Reaction | Rate |
| --- | --- |
| SKHpt → SKPHpt | k_1_ × SKHpt |
| SKPHpt → SKHpt | k_2_ × SKPHpt |
| SKPHpt + RR1 → SKPHptRR1 | k_3_ × SKPHpt × RR1 |
| SKPHptRR1 → SKPHpt + RR1 | k_4_ × SKPHptRR1 |
| SKPHptRR1 → SKHptRR1P | k_5_ × SKPHptRR1 |
| SKHptRR1P → SKHpt + RR1P | k_6_ × SKHptRR1P |
| SKHpt + RR1P → SKHptRR1P | k_7_ × SKHpt × RR1P |
| SKPHptRR1 → SKHptRR1 | k_8_ × SKPHptRR1 |
| SKHptRR1 → SKHpt + RR1 | k_9_ × SKHptRR1 |
| SKHpt + RR1 → SKHptRR1 | k_10_ × SKHpt × RR1 |
| RR1P → RR1 | K_11_ × RR1P |
| RR1P + SKHpt →RR1PSKHpt | k_12_ × SKHpt × RR1P |
| RR1PSKHpt → RR1P+SKHpt | k_13_ × RR1PSKHpt |
| RR1PSKHpt → RR1SKHptP | k_14_ × RR1PSKHpt |
| RR1SKHptP → RR1PSKHptP | K_24_ × RR1SKHptP |
| RR1SKHptP → RR1 + SKHptP | k_15_ × RR1SKHptP |
| RR1 + SKHptP → RR1SKHptP | k_16_ × RR1 × SKHptP |
| SKHptP → SKHpt | k_17_ × SKHptP |
| SKHptP + RR2 → SKHptPRR2 | k_18_ × SKHptP × RR2 |
| SKHptPRR2 → SKHptP + RR2 | k_19_ × SKHptPRR2 |
| SKHptPRR2 → SKHptRR2P | k_20_ × SKHptPRR2 |
| SKHptRR2P → SKHptPRR2 | k_25_ × SKHptRR2P |
| SKHptRR2P → SKHpt + RR2P | k_21_ × SKHptRR2P |
| SKHpt + RR2P → SKHptRR2P | k_22_ × SKHpt×RR2P |
| RR2P → RR2 | k_23_ × RR2P |

### Architecture M3

| Reaction | Rate |
| --- | --- |
| SKRR1Hpt → SKPRR1Hpt | k_1_ × SKRR1Hpt |
| SKPRR1Hpt → SKRR1Hpt | k_2_ × SKPRR1Hpt |
| SKPRR1Hpt → SKRR1PHpt | k_5_ × SKPRR1Hpt |
| SKPRR1Hpt → SKRR1Hpt | k_8_ × SKPRR1Hpt |
| SKRR1PHpt → SKRR1Hpt | K_11_ × SKRR1PHpt |
| SKRR1PHpt → SKRR1HptP | k_14_ × SKRR1PHpt |
| SKRR1HptP → SKRR1PHpt | K_24_ × SKRR1HptP |
| SKRR1HptP → SKRR1Hpt | k_17_ × SKRR1HptP |
| SKRR1HptP + RR2 → SKRR1HptPRR2 | k_18_ × SKRR1HptP × RR2 |
| SKRR1HptPRR2 → SKRR1HptP + RR2 | k_19_ × SKRR1HptPRR2 |
| SKRR1HptPRR2 → SKRR1HptRR2P | k_20_ × SKRR1HptPRR2 |
| SKRR1HptRR2P → SKRR1HptPRR2 | k_25_ × SKRR1HptRR2P |
| SKRR1HptRR2P → SKRR1Hpt + RR2P | k_21_ × SKRR1HptRR2P |
| SKRR1Hpt + RR2P → SKRR1HptRR2P | k_22_ × SKRR1Hpt×RR2P |
| RR2P → RR2 | k_23_ × RR2P |

### Architecture M4

| Reaction | Rate |
| --- | --- |
| SKRR1HptRR2 → SKPRR1HptRR2 | k_1_ × SKRR1HptRR2 |
| SKPRR1HptRR2 → SKRR1HptRR2 | k_2_ × SKPRR1HptRR2 |
| SKPRR1HptRR2 → SKRR1PHptRR2 | k_5_ × SKPRR1HptRR2 |
| SKPRR1HptRR2 → SKRR1HptRR2 | k_8_ × SKPRR1HptRR2 |
| SKRR1PHptRR2 → SKRR1HptRR2 | K_11_ × SKRR1PHptRR2 |
| SKRR1PHptRR2 → SKRR1HptPRR2 | k_14_ × SKRR1PHptRR2 |
| SKRR1HptPRR2 → SKRR1PHptRR2 | K_24_ × SKRR1HptPRR2 |
| SKRR1HptPRR2 → SKRR1HptRR2 | k_17_ × SKRR1HptPRR2 |
| SKRR1HptPRR2 → SKRR1HptRR2P | k_20_ × SKRR1HptPRR2 |
| SKRR1HptRR2P → SKRR1HptPRR2 | k_25_ × SKRR1HptRR2P |
| SKRR1HptRR2P → SKRR1HptRR2 | k_23_ × SKRR1HptRR2P |

## Parameter values and protein abundances for the mass action mathematical models

The parameter values for the mass action models were generated by combining all possible values described in Table S4. The protein abundances were approximated to the orders of magnitudes experimentally determined in Table S3 and then also combined using a latin hypercube approach.

## Mathematical Models for the Spo0 Phosphorelay

### Parameters and abundances for the Spo0 system in *Bacillus subtilis* compiled from ^67,100^

| Reaction | Rate constants | |
| --- | --- | --- |
| SK → SKP | 0.0019 s^-1^ | |
| SKP → SK | 0 | |
| SK+RR → SKRR | 100 | |
| SKRR→SK + RR | 200 | |
| SKP+RR → SKPRR | 200 | |
| SKPRR→SKP + RR | 100 | |
| SKPRR→SKRR | 0 | |
| SKPRR → SKRRP | 0.08 s^-1^ | |
| SKRRP → SKRR | 0 | |
| SKRRP → SK + RRP | 100 | |
| SK + RRP → SKRRP | 200 | |
| RRP → RR | 0.05 | |
| RRP + Hpt → RRPHpt | 100 | |
| RRPHpt → RRP + Hpt | 2300 | |
| RRPHpt →RRHptP | 1.5*10^-5^ s^-1^ | |
| RRHptP → RR + HptP | 2300 | |
| RR + HptP → RRHptP | 100 | |
| HptP + RR2 → HptPRR2 | 400 | |
| HptPRR2 → HPtP + RR2 | 100 | |
| HptPRR2 → HptRR2P | 0.20 s^-1^ | |
| HptRR2P → Hpt + RR2P | 100 | |
| Hpt + RR2P → HptRR2P | 400 | |
| RR2P → RR2 | 0.0001-0.006 | |
|  |  | |
|  |  | |
|  |  | |
|  |  | |
|  |  | |
| PR proteins | Abundances | |
| KinA | 610 molecules cell ^-1^ | |
| Spo0F | 123 molecules cell ^-1^ | |
| Spo0A | 192 molecules cell ^-1^ | |
| Spo0B | 267 molecules cell ^-1^ | |
|  |  | |
|  |  | |
| Mathematical Models for the Sln1 Phosphorelay | |  |

### Parameter values and abundances for the Sln1-Ypd1-Ssk1-Skn7 system in *Sacharomyces cerevisiae* compiled from ^42,43^

| Reaction | Sln1:Ypd1:Ssk1 | Sln1:Ypd1:Skn7 |
| --- | --- | --- |
| SKRR → SKPRR | 10000 | 10000 |
| SKPRR → SKRR | 100 | 100 |
| SKPRR → SKRRP | 10000 | 10000 |
| SKRRP→SKRR | 0.001  (0.0005 w/osmolites) | 0.001 |
| SKRRP + Hpt → SKRRPHpt | 2000 M^-1^ s^-1^ | 2000 M^-1^ s^-1^ |
| SKRRPHpt → SKRRP + Hpt | 2800 s^-1^ | 2800 s^-1^ |
| SKRRPHpt →SKRRHptP | 29 s^-1^ | 29 s^-1^ |
| SKRRHptP →SKRRPHpt | 7.5 s^-1^ | 7.5 s^-1^ |
| SKRRHptP → SKRR + HptP | 14000 | 14000 |
| SKRR + HptP → RRHptP | 2000 | 2000 |
| HptP + RR2 → HptPRR2 | 2000 | 2000 |
| HptPRR2 → HPtP + RR2 | 4800 | 2800 |
| HptPRR2 → HptRR2P | 160 s^-1^ | 1.4 s^-1^ |
| HptRR2P → HptPRR2 | 0 | 0.4 s^-1^ |
| HptRR2P → Hpt + RR2P | 0 | 10000 |
| Hpt + RR2P → HptRR2P | 0 | 2 |
|  |  |  |
|  |  |  |
| PR Proteins | Protein abundances |  |
| Sln1 | 696 molecules cell ^-1^ |  |
| Ypd1 | 6560 molecules cell ^-1^ |  |
| Ssk1 | 1018 molecules cell ^-1^ |  |
| Skn7 | 2536 molecules cell ^-1^ |  |
|  |  |  |

## Detailed description of the simulations performed for each system

### Identifying equivalent protein concentrations for the Spo0 and Sln1 phosphorelays

Identifying the most equivalent response curves between the cognate architecture and the alternatives was done using deterministic simulations in the following way.

First, and for the Spo0 phosphorelay, we created a signal-response curve for the cognate architecture by systematically changing the potential signals (k1, k18 and k25) and calculating the steady state of the circuit at each set signal intensities. k1 and k18 were scanned simultaneous and independently between 10^-6^ and 10^4^. Scanning was done in logarithmic space, with four uniformly spaced intervals per decade. 1600 simulations were done.

Then, for each alternative architecture, we performed the same 1600 simulations and scanned the concentrations of proteins that add been fused or divided with respect to the native architecture by one order of magnitude above and below the native amounts. The scanning was done uniformly in logarithmic space, with 20 sampling intervals per decade. Thus, we ran between 64000 and 106 400 000 million simulations per alternative architecture.

Then, we used a square minimum criterion to identify the protein concentrations that led to the smallest aggregate differences in the signal response curve.

### Calculating physiological variables for the alternative architectures

#### Cost

Once we add the most equivalent systems we calculated the cost for the proteins in those systems and compared that cost to that of the native system as described in the main text.

#### Logarithmic gains and sensitivities

Logarithmic gains were calculated for the basal states of the alternative architectures for the same system.

Sensitivities to individual parameters were calculated for the basal state using the formula from the main text.

#### Response times

Response times were calculated for each PR circuit in two sets of in silico experiments. In the first set, we started with the native architecture fully unphosphorylated and scanned k1 and k2, simultaneous and independently, between 10^-6^ and 10^4^. Scanning was done in logarithmic space, with four uniformly spaced intervals per decade. 1600 simulations were done to calculate the steady state of the system. Then, 1600 additional simulations were done to follow the time course of the dependent variables from a fully unphosphorylated state to a state where all variables were within 0.90 of its steady state concentrations. This was repeated for each alternative architecture, making for a total of 16000 simulations per PR.

In the second set of experiments, we started with the native architecture under saturating phosphorylation and scanned k1 and k2, simultaneous and independently, between 10^-6^ and 10^4^. Scanning was done in logarithmic space, with four uniformly spaced intervals per decade. 1600 simulations were done to calculate the steady state of the system. Then, 1600 additional simulations were done to follow the time course of the dependent variables from a fully phosphorylated state to a state where all variables were within 0.90 of its steady state concentrations. This was repeated for each alternative architecture, making for a total of 16000 simulations per PR. Overall, we performed 32000 simulations for the Spo0 and Sln1 PR.

#### Calculating information transmission

To calculate information transmission we performed the same set of experiments described for the response times, solving the systems numerically with a Gillespie algorithm.

Because Gillespie algorithms are significantly slower than deterministic algorithms, we initially performed 1600 simulations for the native architecture as described in response times. We then calculated mutual information applying the formula described in the main text. We looked at the instantaneous information being transmitted through an architecture from the moment we shifted its signal until it reached 80% of the new steady state. Then we looked at the information being transmitted through the architecture after achieving the new steady state. We found that steady state information transmission was always higher than instantaneous information transmitted. Because of this observation, and save computational resources and time we focused on steady state information transmission.

First, we converted the deterministic steady state concentrations into number of molecules. Then, we ran twenty five Gillespie simulations for each steady state during 20 minutes and calculated information transmission as described in the main text. Overall, we ran 800 000 simulations to calculate information for each architecture in the Spo0 and Sln1 systems.

Then, for calculating the information transmission for the Atlas shown in Table S1 we performed 200 000 simulations per set of concentrations (40000 simulations per PR architecture) and we tested 39 sets of concentrations, for a total of 7 800 000 simulations.
