# Supplementary material for: Maximization of information transmission influences selection of native phosphorelay architectures
Source: PeerJ. 2021 Jun 10;9:e11558. doi: 10.7717/peerj.11558 (PMC8199921; doi:10.7717/peerj.11558)
Supplement: Supplemental Information 2 [file peerj-09-11558-s002.pdf]

Table S2 – Phosphorelay circuits for which there is abundance information.

Organism (average cell volume in μm^3)	Phosphorelay	Protein abundances (ppm)	Estimated number of proteins per cell[1]	Ratios of abundance (order of magnitude)
Saccharomyces cerevisiae (~ 70 [79])	Sln1:Ypd1:Ssk1 HKRR:Hpt:RR	151:188:177 [80]	66000: 82000: 77000	1:1:1:1
	Sln1:Ypd1:Skn7 HKRR:Hpt:RR	151:188:175 [80]	66000: 82000: 77000	1:1:1:1
Schizosaccharomyces pombe (~ 100 [81,82])	Mak1:Mpr1:Mcs4 HKRR:Hpt:RR	44:62:470 [83]	11000: 39000: 293000	1:1:1:10
	Mak2:Mpr1:Mcs4 HKRR:Hpt:RR	53:62:470 [83]	33000: 39000: 293000	1:1:1:10
	Mak3:Mpr1:Mcs4 HKRR:Hpt:RR	53:62:470 [83]	33000: 39000: 293000	1:1:1:10
Cryptococcus neoformans (~ 50-70 [84])	CNH02350 HKRRHptRR	2.63 [85]	980	1:1:1:1
	CNC03340:CNM01530:CNB03090 HKRR:Hpt:RR	42:367:14 [85]	16000: 137000: 5000	10:10:100:1
	CNA03400:CNM01530:CNB03090 HKRR:Hpt:RR	3:367:14 [85]	1000: 137000: 5000	1:1:100:1

[1] This number is calculated by multiplying cell volume (μm^3) by the average number of proteins in cells per μm^3 times the protein abundance in parts per million:

$$\frac{Cell\ Volume \times 6.23\times 10^{6} \times Protein\ abundances}{10^{6}}$$

Organism (average cell volume in μm³)	Phosphorelay	Protein abundances (ppm)	Estimated number of proteins per cell[2]	Ratios of abundance (order of magnitude)
Arabidopsis thaliana (2000 [86])	AKH2:AHP1-AHP4<br>HKRRRR:Hpt	0.15:8-50 [87]	2000: 99000-623000	1:1:10:1<br>1:1:100:1
	AKH3:AHP1-AHP4<br>HKRRRR:Hpt	1: 8-50 [87]	12000: 99000-623000	1:1:10:1<br>1:100:1:1
	AKH4:AHP1-AHP4<br>HKRRRR:Hpt	0.62: 8-50 [87]	8000:99000-623000	1:1:10:1<br>1:1:100:1
	AHK1:AHP2-AHP3:ARR1-3,5,7,9,14,16<br>HKRR:Hpt:RR	0.3:15-50:0.01-2 [87]	4000: 187000-623000:100-25000	1:1:100:1<br>1:1:100:10<br>10:10:1000:1
	AHK5:AHp1-3, AHP5-6:ARR1-2<br>HKRR:Hpt:RR	0.1:9-50:2 [87]	1200: 112000-623000: 25000	1:1:100:10
	ETR1:AHP5:RR1-2<br>HKRR:Hpt:RR	2.5:0.02:2 [87]	31000: 250-623000: 25000	1:1:1:1<br>10:10:1:10

[2] This number is calculated by multiplying cell volume (μm³) by the average number of proteins in cells per μm³ times the protein abundance in parts per million: $\frac{Cell\ Volume\ \times 6.23 \times 10^6 \times Protein\ abundances}{10^6}$

Organism (average cell volume in μm³)	Phosphorelay	Protein abundances (ppm)	Estimated number of proteins per cell[3]	Ratios of abundance (order of magnitude)
	TorS:TorR<br>HKRRHpt:RR	0.34:6 [90,91]	212:3728	1:1:1:10
	EvgS:EvgA<br>HKRRHpt:RR	0.31:88 [90,91]	193:54800	1:1:1:100
Escherichia coli<br>(~ 0.1-3 [88,89])	BarA:UvrY<br>HKRRHpt:RR	3.5:55 [90,91]	2180:34265	1:1:1:10
	ArcB:ArcA<br>HKRRHpt:RR	51:1065 [90,91]	30:6600	1:1:1:10
	RcsC:RcsD:RcsB<br>HKRR:Hpt:RR	3.:7.4:375 [90,91]	19:46: 2340	1:1:1:100
Shigella flexneri<br>(~ 0.1-3 [88,89])	BarA:UvrY<br>HKRRHpt:RR	3.94:8.74 [92,93]	24:54	1:1:1:1
	ArcB:ArcA<br>HKRRHpt:RR	34.9:1094	220:.6800	1:1:1:100
Pseudomonas aeruginosa<br>(~ 0.5 [94])	PA0413:PA0416<br>HKRRHpt:RR	158:BDL [91,95]	580:-	1:<1
	PA4112<br>HKRRHptRR	7.2[91,95]	26	1:1:1:1
Shewanella oneidensis<br>(1-4 [96])	SO3207:SO3206:SO3209<br>HKHpt:RR:RR	452:17.1:768 [91]	2800: 110: 4800	10:1:10:10<br>10:1:10:100
	SO0859:SO0860<br>HKRRHpt:RR	88:79 [91]	550:490	1:1:1:1
Desulfovibrio vulgaris<br>(2.4 [97])	DVU_3062:DVU_3061<br>HKRRHpt:RR	80:79 [98,99]	1200:1200	1:1:1:1

[3] These numbers are calculated by multiplying cell volume (μm³), average number of proteins in cells per μm³, and the protein abundance in parts per million:

$$\frac{Cell\ Volume \times 6.23\times10^{6}\times Protein\ abundances}{10^{6}}$$

Organism (average cell volume in μm^3)	Phosphorelay	Protein abundances (ppm)	Estimated number of proteins per cell[4]	Ratios of abundance (order of magnitude)
Salmonella enterica (0.8 [94])	RcsC:RcsD:RcsB HKRR:Hpt:RR	3.4:3.9:327 [91]	17:19:1600	1:1:1:100
Bacillus subtilis (1.5 [94])	KinA:Spo0F:Spo0B:Spo0A HK:RR:Hpt:RR	1.3:446:12.4:185 [91]	12:4200:110:1700	1:100:1:100
	KinB:Spo0F:Spo0B:Spo0A HK:RR:Hpt:RR	10:446:12.4:185 [91]	93:4200:110:1700	1:100:1:100
	KinC:Spo0F:Spo0B:Spo0A HK:RR:Hpt:RR	8.8:446:12.4:185 [91]	82:4200:110:1700	1:100:1:100

BDL: Below detection limit

[4] These numbers are calculated by multiplying cell volume (μm^3), average number of proteins in cells per μm^3, and the protein abundance in parts per million:

$$\frac{Cell\ Volume \times 6.23 \times 10^6 \times Protein\ abundances}{10^6}$$
