# Supplementary material for: Maximization of information transmission influences selection of native phosphorelay architectures
Source: PeerJ. 2021 Jun 10;9:e11558. doi: 10.7717/peerj.11558 (PMC8199921; doi:10.7717/peerj.11558)
Supplement: Supplemental Information 3 [file peerj-09-11558-s003.pdf]

Table S3 – Numerical values of the rate constants of the reactions in the PR models used in the stochastic simulations, represented in Figure 4. These values are based on [34]. We found a range of variation in four of the rate constants (k1, k2, k10 and k19) [38–40,50]. For the generality of the results, we used both the minimum and the maximum value of these four rate constants, obtaining 16 different combinations of numerical values. Each one of these 16 combinations of rate constant values was used in each one of the 5 PR models to obtain 100 stochastic trajectories.

Rate Constant	Experimental values	Scanning	Type of scanning
k1	Min= 0.002 s^{-1}<br>Middle = 0.028 s^{-1}<br>Max = 20 s^{-1}	Yes	Continuous, from 10^{-4} s^{-1} to 10^{3} s^{-1}
k2, k24k k25	Min= 0 s^{-1}<br>Max = 0.00055 s^{-1}	Yes	Continuous, from 10^{-4} s^{-1} to 10^{3} s^{-1}
k3, k8, k11, k14, k17	0.000139 nM^{-1} s^{-1}	No	–
k4	0.083 s^{-1}	No	–
k5	0.083 s^{-1}	No	–
k9, k18	0.028 s^{-1}	No	–
k10, k19	Min=0.0056 s^{-1}<br>Max=0.028 s^{-1}	Yes	Discrete, with all possible combinations of k10 and k19 and $k10 \in \{0.0056, 0.028\}$ and $k19 \in \{0.0056, 0.028\}$
k12	0.139 s^{-1}	No	–
k13, k16	0.22 s^{-1}	No	–
k15	0.056 s^{-1}	No	–
